# The presentation, management and outcome of patients with ductal carcinoma in situ (DCIS) with microinvasion (invasion ≤1 mm in size)—results from the UK Sloane Project

**DOI:** 10.1038/s41416-022-01983-4

**Published:** 2022-10-12

**Authors:** Abeer M. Shaaban, Bridget Hilton, Karen Clements, David Dodwell, Nisha Sharma, Cliona Kirwan, Elinor Sawyer, Anthony Maxwell, Matthew Wallis, Hilary Stobart, Senthurun Mylvaganam, Janet Litherland, Samantha Brace-McDonnell, Joanne Dulson-Cox, Olive Kearins, Elena Provenzano, Ian O. Ellis, Sarah E. Pinder, Alastair M. Thompson

**Affiliations:** 1 Queen Elizabeth Hospital Birmingham and University of Birmingham, Birmingham, UK; 2 NHS England and NHS Improvement, Birmingham, UK; 3 Nuffield Department of Population Health, University of Oxford, Oxford, UK; 4 Leeds Teaching Hospitals NHS Trust, Leeds, UK; 5 Division of Informatics, Imaging & Data Sciences. School of Health Sciences, Faculty of Biology, Medicine and Health, University of Manchester, Manchester, M13 9PT UK; 6 School of Cancer and Pharmaceutical Sciences, King’s College London, Guy’s Comprehensive Cancer Centre at Guy’s and St Thomas’ Hospitals NHS Foundation Trust, London, UK; 7 Addenbrookes Hospital, Cambridge and Cambridge Breast Unit, and NIHR Cambridge Biomedical Research Centre, Cambridge University Hospitals NHS Trust, Cambridge, UK; 8 Independent Cancer Patients’ Voice, London, UK; 9 Royal Wolverhampton Hospital NHS Trust, Wolverhampton, UK; 10 NHS Greater Glasgow and Clyde, Glasgow, UK; 11 Nottingham University Hospitals, Nottingham, UK; 12 Nottingham Breast Cancer Research Centre, Division of Cancer and Stem Cells, School of Medicine, Nottingham City Hospital, The University of Nottingham, Nottingham, UK; 13 Baylor College of Medicine, Houston, TX USA

**Keywords:** Breast cancer, Cancer epidemiology

## Abstract

**Background:**

The diagnosis, management and prognosis of microinvasive breast carcinoma remain controversial.

**Methods:**

We analysed the outcomes of patients with DCIS with and without microinvasion diagnosed between 2003 and 2012 within the Sloane project.

**Results:**

Microinvasion was recorded in 521 of 11,285 patients (4.6%), with considerable variation in reported incidence among screening units (0–25%). Microinvasion was associated with high-grade DCIS, larger DCIS size, comedo necrosis and solid, cribriform architecture (all *P* *<* 0.001). Microinvasion was more frequent in patients who underwent mastectomy compared with breast-conserving surgery (BCS) (6.9% vs 3.6%, *P* *<*  0.001), and in those undergoing axillary nodal surgery (60.4% vs 30.3%, *P* *<*  0.001) including the subset undergoing BCS (43.4% vs 8.5%, *P* *<* 0.001). Nodal metastasis rate was low and not statistically significant difference from the DCIS only group (*P* = 0.68). Following median follow-up of 110 months, 3% of patients had recurrent ipsilateral high-grade DCIS, and 4.2% developed invasive carcinoma. The subsequent ipsilateral invasion was of Grade 3 in 71.4% of patients with microinvasion vs 30.4% in DCIS without microinvasion (*P* = 0.02). Distant metastasis and breast cancer mortality were higher with microinvasion compared with DCIS only (1.2% vs 0.3%, *P* = 0.01 and 2.1% vs 0.8%; *P* = 0.005).

**Conclusions:**

The higher breast cancer mortality with microinvasion indicates a more aggressive disease.

## Introduction

Microinvasion, defined as one or more foci of invasion of ≤1 mm in size, is predominantly identified in association with high-grade ductal carcinoma in situ (DCIS). However, it can also be seen with other DCIS grades, lobular carcinoma in situ (LCIS) and Paget’s disease [1].

In 1995, microinvasion was categorised by the UK National Coordinating Committee for Breast Pathology as a focus of invasion 1 mm or less identified within the non-specialised stroma. Based on this criterion, small foci of invasion measuring less than 1 mm but localised to the specialised loose stroma within lobules were not interpreted as microinvasion. This stromal feature was subsequently dropped from the histological definition, partly because of the subsequent uncertainty of how to classify lesions within the specialised stroma (if not as microinvasion) and partly due to the difficulty in distinguishing specialised from non-specialised stroma, especially in the context of high-grade DCIS that is often associated with dense chronic inflammation [2].

The accurate incidence of microinvasion is difficult to ascertain, but it is estimated that it can be seen in ~5–10% of DCIS cases and comprises around 1% of all breast cancers [3]. The Surveillance, Epidemiology and End Results (SEER) registry data reported microinvasion in 3.2% out of a total of 134,569 women registered [4].

Controversy also exists as to the natural history of microinvasive disease and its impact on outcomes. Whether microinvasion behaves, and should be managed, as DCIS or whether it represents true, albeit small, invasive disease is debated. Moreover, there is no consensus as to whether there is a role for routine axillary staging if a diagnosis of microinvasion has been made. For example, one group has recommended lymph-node sampling in patients with DCIS with microinvasion, but this was based on a series of 51 DCIS patients of whom only 6 had microinvasion; 5 had nodal involvement, including 3 patients from the microinvasion group [5]. There are, however, no specific national or international guidelines on whether or not axillary lymph-node examination should be performed in patients with microinvasion, reflecting the paucity of data.

Large, well-annotated cohorts with long-term follow-up are therefore needed to address these clinically relevant issues. Following our report of the pathological features of pure DCIS [6, 7], we aimed to analyse the natural history, management and outcome of DCIS with microinvasive carcinoma diagnosed by either preoperative core biopsy or at surgical excision within the UK Sloane Project, a prospective cohort of screen-detected non-invasive breast cancer with long-term follow-up.

## Methods

The Sloane Project is a UK prospective cohort study of screen-detected non-invasive breast neoplasia governed by NHS England and NHS Improvement (previously Public Health England, PHE). DCIS lesions, including those with microinvasion, diagnosed between 2003 and 2012 were submitted from UK NHS Breast Screening Programme Units. Comprehensive imaging, surgical, pathology and oncology data were collected at screening unit and hospital levels and submitted to the Sloane Project team, who entered information into a secure database. All participating units followed the Sloane Project protocols [8] and the National Health Service Breast Screening Programme (NHSBSP) guidelines for pathology reporting, including DCIS cytonuclear grading, definitions of atypia, microinvasion, comedo necrosis and assessment of surgical margins [2].

Pathologists were also required to participate in the National Breast External Quality Assurance Scheme. Follow-up data, including subsequent events occurring 6 months or more from the initial diagnosis, and patient survival were collected from local data and cross-referenced against national registries to ensure accuracy [6, 9]. For patient outcome data from England, the following national datasets were cross-checked: English Cancer Analysis System (CAS), Hospital Episode Statistics (HES), Cancer Waiting Times (CWT), English National Radiotherapy Dataset (RTDS), Systemic Anti-Cancer Therapy dataset (SACT), Office for National Statistics (ONS) mortality data, Mortality and Birth Information System (MBIS).

The methodology of the Sloane project data collection and verification is described in detail elsewhere (Clements et al., manuscript submitted).

## Results

Of a total of 11,285 DCIS cases included, microinvasion was identified in 512 (4.6%). There was no significant difference in the incidence of microinvasion by patient age; 4.7% and 4.6% of patients under and over the age of 50, respectively, had microinvasion recorded, with an incidence of 4.2% and 4.9% of patients under and over the age of 60, respectively.

The reported incidence of microinvasion decreased from 7% in 2003/2004 to 3% in 2011/2012 with an overall incidence over the study period of 5% (Table 1). However, marked variation in the reported incidence of microinvasion in those patients submitted to the Project was noted amongst contributing screening units (0–25%).

**Table 1:** The reported incidence of microinvasion by screening year.

Screening year	Microinvasion present	%	Microinvasion absent	%	Unknown	%	Total
03/04	74	7%	1040	93%	3	0%	1117
04/05	80	6%	1210	94%	1	0%	1291
05/06	57	4%	1260	95%	3	0%	1320
06/07	73	5%	1290	95%	2	0%	1365
07/08	54	4%	1282	95%	17	1%	1353
08/09	62	5%	1245	95%	9	1%	1316
09/10	42	3%	1272	97%	4	0%	1318
10/11	49	4%	1224	96%	3	0%	1276
11/12	30	3%	941	96%	10	1%	981
**Grand total**	**521**	**5%**	**10,764**	**95%**	**52**	**0%**	**11,337**

### Clinicopathological features associated with microinvasion

Microinvasion was significantly associated with high cytonuclear grade of DCIS; it was reported in 5.9% of 7182 cases of high-grade DCIS compared to 2.9% of 3093 intermediate grade and <1% of 995 low-grade DCIS (*P* < 0.001). Microinvasion was associated with larger DCIS lesions (*P* < 0.001) being diagnosed in 2.2% of DCIS less than 10 mm in size, 3.9% of DCIS 10–20 mm, 5.8% of DCIS 20–30 mm, 5.2% of DCIS 30–40 mm and 8.0% of DCIS > 40 mm. It was also associated with the presence of comedo necrosis (*P* < 0.001), and solid (*P* < 0.001), cribriform (*P* < 0.001) or flat (*P* = 0.03) DCIS architectures (Table 2).

**Table 2:** The relation between microinvasion and clinicopathological and treatment parameters.

	DCIS with microinvasion		DCIS without microinvasion		Total	*P* value
DCIS grade	521	4.6%	10,764	95.4%	11,285	
Low	7	0.7%	988	99.3%	995	
Intermediate	91	2.9%	3002	97.1%	3093	
High	421	5.9%	6761	94.1%	7182	
Unknown	2	13.3%	13	86.7%	15	<0.001
Size of DCIS	521	4.6%	10,764	95.4%	11,285	
<10 mm	64	2.2%	2809	97.8%	2873	
10–20 mm	124	3.9%	3053	96.1%	3177	
20–30 mm	115	5.8%	1863	94.2%	1978	
30–40 mm	55	5.2%	1011	94.8%	1066	
>40 mm	158	8.0%	1829	92.0%	1987	
No residual DCIS*	0	0.0%	93	100.0%	93	
Unknown	5	4.5%	106	95.5%	111	<0.001
Treatment	521	4.6%	10,764	95.4%	11,285	
BCS only	44	1.5%	2908	98.5%	2952	
BCS + RT	230	5.0%	4325	95.0%	4555	
BCS (unknown RT)	14	3.6%	378	96.4%	392	
Mx	232	6.9%	3140	93.1%	3372	
No surgery	1	7.1%	13	92.9%	14	<0.001
RT (BCS only)	230	5.0%	4325	95.0%	4555	
No RT (BCS only)	44	1.5%	2908	98.5%	2952	<0.001
BCS, margin width	288	3.6%	7611	96.4%	7899	
<2 mm	52	4.8%	1025	95.2%	1077	
2 mm or more	217	3.4%	6153	96.6%	6370	
Unknown	19	4.2%	433	95.8%	452	0.06
Axillary nodes examined	521	4.6%	10,764	95.4%	11,285	
Yes	332	9.8%	3070	90.2%	3402	
No	184	2.4%	7587	97.6%	7771	
Unknown	5	4.5%	107	95.5%	112	<0.001
Axillary nodes examined (BCS patients only)	288	3.6%	7611	96.4%	7899	
Yes	125	18.7%	545	81.3%	670	
No	161	2.2%	6999	97.8%	7160	
Unknown	2	2.9%	67	97.1%	69	<0.001

*DCIS fully excised on diagnostic biopsy.

### Surgery and adjuvant therapy

Microinvasion was identified more frequently in patients who underwent mastectomy (6.9%) compared with those who had breast-conserving surgery (BCS) (3.6%; *P* < 0.001). In patients who underwent BCS, there was no significant association between the presence of microinvasion and margin status or margin width (Table 2).

Axillary surgery was performed in 3406 patients (Table 2). This included sentinel lymph node (SLN) in 1533 patients, SLN and sampling (*n* = 170), axillary node sampling (*n* = 1570), SLNB and clearance (ANC, *n* = 11) and ANC (*n* = 114). The latter (ANC) group was mainly patients who underwent mastectomy (*n* = 103). The percentage of ANC and mastectomy in the microinvasion patients declined from 7.9% at the start of the project in 2003/2004 to 1.4% in 2011/2012.

Unfortunately, data on whether the diagnosis of microinvasion was made on core biopsy or subsequent excision is not available; therefore, it was not possible to ascertain the proportion of patients who underwent nodal surgery at the first operation as a result of a preoperative diagnosis of microinvasion. However, axillary nodal surgery was more commonly performed in patients diagnosed with DCIS with microinvasion compared to those without (60.4% compared to 30.3%). This remained the case when the analysis was restricted to patients who underwent BCS. In patients who had BCS as the initial surgery, axillary node surgery was more frequently performed as subsequent surgery in patients with microinvasive carcinoma (38.4% for microinvasion vs 7.9% for DCIS alone, *P* < 0.001) (Table 3).

**Table 3:** Axillary nodal surgery for DCIS with and without microinvasion for all patients, and the subset that underwent breast-conserving surgery only.

	DCIS with microinvasion		DCIS without microinvasion		Total		*P* value
	*N*	%	*N*	%	*N*	%	
Axillary nodes examined at:							
First surgery/before first surgery*	232	69.9%	2466	80.3%	2698	79.3%	<0.001
Subsequent surgery	100	30.1%	604	19.7%	704	20.7%	
Axillary nodes examined (BCS patients only) at:							
First surgery/before first surgery*	77	61.6%	502	92.1%	579	86.4%	<0.001
Subsequent surgery	48	38.4%	43	7.9%	91	13.6%	

^*^Upfront sentinel node before breast surgery.

The rate of nodal metastasis at first nodal surgery was, however, very low and not statistically significantly different between those with and without microinvasion (2/521, 0.4% and 10/10764, 0.1%, respectively, *P* = 0.27).

Patients with microinvasion who underwent BCS were more likely to receive radiotherapy than those with BCS for pure DCIS (*P* < 0.001) (Table 2).

### Subsequent events

Follow-up data were available for patients from England only (*n* = 9423). These patients were followed up for a maximum of 164 months with a median of 110 months (range 4–164 months). Patients with pure DCIS and those with DCIS plus microinvasion showed a very low event rate with no statistically significant difference between the groups. Of the patients diagnosed with microinvasion and treated by BCS, 6 of 261 (3%) had ipsilateral recurrent DCIS and 11 of 261 (4.2%) developed ipsilateral invasive carcinoma. The corresponding proportions of ipsilateral DCIS recurrence and ipsilateral invasive disease for those with pure (i.e., without microinvasion) were 3.4% (211 of 6262) and 5.6% (349 of 6262), respectively, *P* = 0.39.

All subsequent ipsilateral DCIS recurrences following a primary diagnosis of high-grade DCIS with microinvasion were high grade compared with 102/118 (86.4%) of recurrences following high-grade DCIS without microinvasion (*P* = 0.43). All subsequent contralateral DCIS recurrences (6/6) following a primary diagnosis of DCIS with microinvasion were high-grade compared with 24/39 (61.5%) of recurrences following high-grade DCIS without microinvasion (*P* = 0.06). Furthermore, the majority (71.4%) of the subsequent invasive carcinomas in the same breast were of histological grade 3. For patients with DCIS without microinvasion at initial diagnosis, only 30.4% of the ipsilateral subsequent invasive carcinomas were grade 3 (*P* = 0.02). Interestingly, all contralateral DCIS developing following an initial diagnosis of DCIS with microinvasion were also of high grade compared with 59.3% in patients with DCIS only (*P* = 0.03).

Lesion size was not associated with the frequency of subsequent events in the patients with microinvasion. Of note, in those with DCIS without microinvasion, there were more subsequent events in those with lesions under 2 cm compared with those above 2 cm (*P* = 0.005), possibly reflecting the effect of adjuvant therapy more frequently administered for larger lesions.

Ipsilateral events were similar in frequency in patients with and without microinvasion who underwent BCS, *P* = 0.68 (Table 4).

**Table 4:** Ipsilateral subsequent events and type in patients who underwent breast-conserving surgery (BCS) in England only.

Ipsilateral events (England only, BCS)	DCIS with microinvasion	DCIS without microinvasion	Total	*P* value
Total	261	6262	6523	
Total events	18 (incl 1 unknown+	584 (incl 24 unknown type^+^)	602	0.18
Recurrence as DCIS	6	211	217	0.34
Recurrence as Invasive	11	349	360	0.35
Recurrence as invasive with nodal positivity	1*	50	51	0.46

^*^Micrometastasis (<2 mm).

^+^Unknown: whether in situ or invasive carcinoma.

### Distant metastases

The overall distant metastasis rate was very low (0.4%), with only 46 patients with distant metastases identified. Analysis of these low numbers revealed statistically significantly more frequent distant metastases in those patients with DCIS with microinvasion (6/511, 1.2%) compared to those with pure DCIS (40/10764, 0.3%), *P* = 0.01.

### Patient survival

**Fig. 1:**
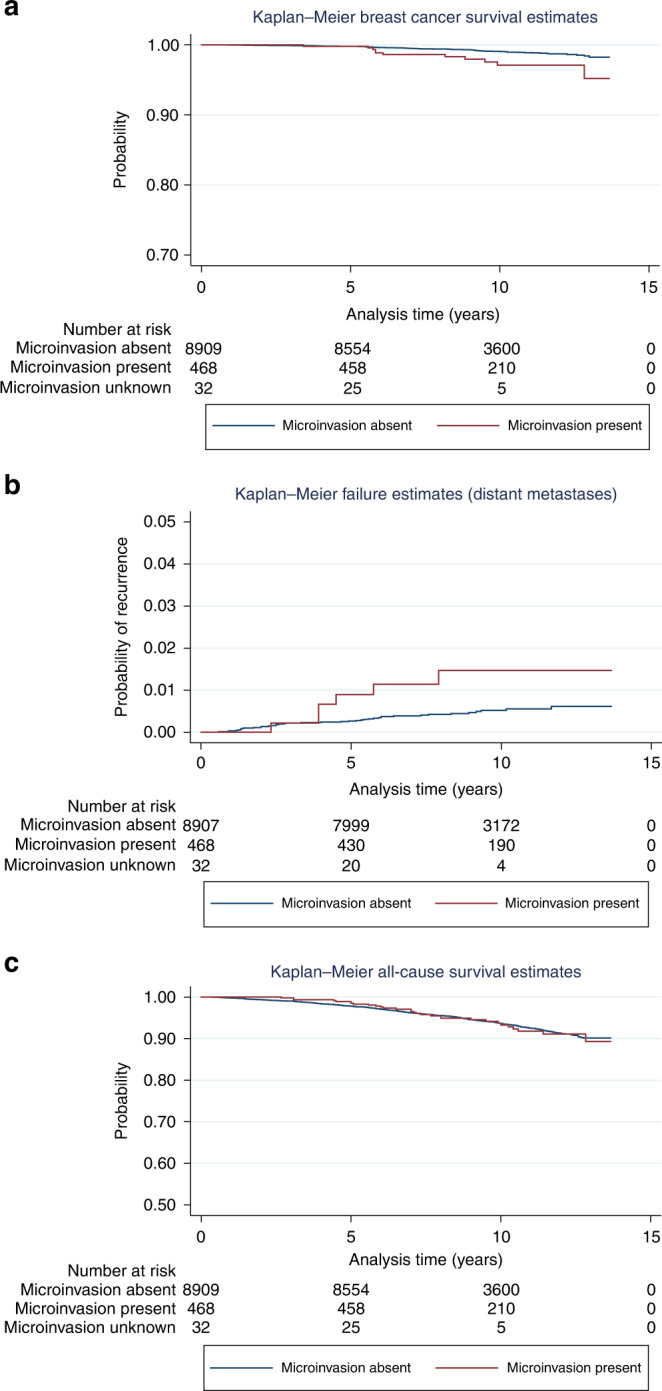
Kaplan Meier survival estimates for patients with DCIS with and without microinvasion (England only). **a** Breast cancer-specific survival (BCSS) for those with and without microinvasion. Log-rank test: *P* value = 0.0027. BCSS is poorer in those with microinvasive disease. **b** Distant metastasis rate for those with and without microinvasion. The rate is very low but patients who presented with DCIS and microinvasion showed significantly poorer overall survival than those who presented with DCIS only. Log-rank test: *P* = 0.02. **c** All-cause mortality for those with and without microinvasion. No statistical difference is seen between those patients with DCIS and without microinvasion. Log-rank test: *P* = 0.94.

Follow-up data were available for patients in England (*n* = 9423). Breast cancer-specific mortality was higher (2.1%) in patients with microinvasion compared with those without it (0.8%). This difference was statistically significant (*P* = 0.005) (Fig. 1a).

Patients who presented with subsequent distant metastasis following an original diagnosis of DCIS plus microinvasion had worse overall survival compared with those with distant metastasis but with initial presentation of DCIS only, Log-rank test, *P* = 0.02 (Fig. 1b). All-cause mortality was not, however, statistically significantly different between patients presenting with and without microinvasive carcinoma, Log-rank test, *P* = 0.94 (Fig. 1c).

The original DCIS lesion size had no effect on survival. When the size was dichotomised using a cut-off value of 2 cm, there was no significant relation between tumour size and patient survival in those with pure DCIS and in patients with microinvasion (log-rank *P* value 0.55 and 0.59, respectively). This persisted when the analysis was limited to high-grade DCIS only (*P* = 0.5 and 0.71, respectively).

## Discussion

We report here on 11,285 patients with screen-detected DCIS specifically examining the features, management and outcome of those 521 with associated microinvasion. The subsequent event rate remained low for both pure DCIS and DCIS with microinvasion; however, the presence of microinvasion was associated with a significantly poorer breast cancer-specific mortality compared to that of patients with DCIS lesions without microinvasion. Moreover, patients with subsequent distant metastases who had an initial diagnosis of DCIS and microinvasive carcinoma had poorer survival than those without microinvasion suggesting that DCIS with microinvasive carcinoma behaves like an invasive cancer with a more aggressive behaviour than pure DCIS.

The largest study to date on microinvasive carcinoma showed similar effects on mortality. A SEER data analysis [10] of 161,394 women with pure DCIS and 13,489 with DCIS and microinvasion reported that the 20-year breast cancer-specific mortality rates were 3.8% and 6.9%, respectively. The rate associated with microinvasion was similar to that of small invasive carcinoma of up to 1 cm (6.8%) and lower than that for women with invasive carcinoma of 1.1–2.0 cm in size (12.1%). However, another, meta-analysis of axillary staging in patients with DCIS and microinvasion, that included 2959 patients from 23 studies, showed a survival rate of patients with microinvasion was overall very similar to those with pure DCIS [11]. In a smaller Chinese cohort, of 359 pure DCIS lesions and 80 DCIS with microinvasion and 31 months of follow-up, Fang et al. reported that those with microinvasion had 3-year disease-free survival (DFS), similar to stage pT1a invasive carcinoma and poorer than pure DCIS but there was no significant difference in the overall survival between the three groups [12]. In another small Singaporean study of 198 DCIS cases, of which only 12 showed microinvasion, Chen et al reported that those patients with DCIS with microinvasion had a statistically significant worse outcome, including shorter recurrence free survival (*P* = 0.01), which persisted on multivariate Cox regression analysis. Those lesions exhibited significantly higher densities of tumour microenvironment cellular infiltrate including CD4, FOXP3, CD163 and PDL-1, potentially indicating a role for the inflammatory immune response in disease progression [13].

In the current cohort, axillary sampling was more frequently performed in the presence of microinvasion (in either preoperative core biopsy or found at surgical excision). It is unfortunate that it is not known if this was a result of microinvasion being identified on preoperative core biopsy or because, for example, the patient presented with large, high-grade DCIS. A meta-analysis of axillary staging in patients with DCIS with microinvasion by Choi et al. reported a 2% rate of nodal macrometastasis, with rates for micrometastases and isolated tumour cell clusters (ITCs) of 2% and 3%. There were significant differences in the likelihood of macrometastasis according to focality of microinvasion (whether focal, focal and multifocal, or multifocal) showed significant differences for macrometastases (*P* = 0.033), but this was not the case for micrometastases or ITCs. The authors concluded that, as axillary staging in microinvasion is unlikely to change patient management, a multidisciplinary approach is preferable to routine axillary staging [11].

The number of microinvasive foci has been shown to be prognostic in other series. In a study of 229 examples of pure DCIS and 264 cases of DCIS with microinvasion (median follow-up of 3.9 years), 0 and 13 showed nodal metastasis, respectively. Lesions with three or more microinvasive foci were significantly associated with nodal positivity (*P* = 0.03) and disease relapse (*P* = 0.05). The relapse-free survival for DCIS with microinvasion and for DCIS only was 95.4% and 99%, respectively [14]. A recent study, of 359 microinvasive carcinomas, showed that the number of microinvasive foci and HER2 positivity indicated a more aggressive disease and suggested that those patients might benefit from systemic therapy [15]. Similar to invasive carcinoma, the presence of multiple microinvasive foci should be stated in the pathology reports, however, their precise number is not currently a mandatory parameter for assessment and recording in the DCIS pathology reporting guidelines [2, 16, 17]. The number of microinvasive foci in any one DCIS lesion was not recorded within the Sloane data, and central pathology review of Sloane histology sections was not performed. We cannot therefore examine the significance of this feature in the current cohort.

Nodal metastasis has been reported to be more frequent in larger DCIS lesions. In one study of 24 patients with DCIS measuring 25 mm or more, microinvasion was identified in 25% of cases. The incidence of microinvasion, invasive carcinoma and nodal metastasis following the diagnosis of DCIS was directly related to tumour size, and the authors recommended axillary node sampling for DCIS lesions measuring 35 mm or more [18]. In the current large series, microinvasion was associated with larger DCIS size but we found no association between DCIS size and contemporaneous nodal metastasis, or recurrence or patient mortality in patients with microinvasion.

Microscopic examination remains the gold standard for diagnosing microinvasion although a few imaging studies have attempted to provide some radiological pointers to the presence of microinvasion. Comparing the imaging features of 94 patients with DCIS and 53 patients with DCIS plus microinvasion, Wang et al. [19] reported that large areas of calcifications and distortion on mammography and/or calcification and increased vascularity on ultrasound were radiologically suggestive of microinvasion.

Most of the published data in the literature is based on a definitive diagnosis of microinvasion diagnosed on surgical excision. Indeed, some would argue that a definitive diagnosis of microinvasive carcinoma cannot be made on a core biopsy, as one cannot be certain that the very small invasive focus in the core is not the edge of a (much) larger invasive lesion. The Memorial Sloan–Kettering Cancer Center published their experience with suspected (*n* = 105) and definite microinvasive cases (*n* = 264) diagnosed on conventional needle core biopsies over a 10-year period (2007–2017) [20]. Lesions were upgraded to true invasion (i.e., >1 mm) on surgical excision in 28% and 35% of cases, respectively. Axillary staging at initial surgery was performed in 77% and 94%, respectively (*P* < 0.001). In the whole cohort when axillary staging was performed, factors predictive of the pN1 stage included young age and large areas of microcalcification (*P* < 0.001 for both). On multivariate analysis, the presence of definite microinvasion in core biopsy significantly increased the risk of nodal metastasis (CI = 1.01–60.7, *P* = 0.04). The rate of nodal metastasis with suspected microinvasion in a core biopsy was very low (<1%) and similar to pure DCIS [20].

From a practical point of view, when very small foci of invasive disease are identified, either in core biopsy samples or surgical excision, further levels are extremely helpful to assess the histology, as the invasive focus may be larger on deeper levels, or foci may join up and thus be categorised as true invasive carcinoma. Conversely, it may be clearer that the focus is actually cancerisation of lobules (or of a sclerosing lesion) rather than true invasion. A panel of immunohistochemistry for myoepithelial markers can be helpful to confirm invasion, while noting that the myoepithelial layer around DCIS, with or without microinvasion, can be discontinuous/attenuated. However, when multiple small foci of invasion are seen, one of the pathological challenges is the lack of guidance on whether foci that are close together should be added to represent one single (invasive) tumour or multiple microinvasive foci. In this context, deeper levels can again be valuable to assess if the foci join up or increase in size.

While most pure DCIS lesions are hormone receptor-positive, lesions with microinvasion are reportedly more often ER negative and HER2-positive. In a study of 289 DCIS lesions that included 88 with microinvasion, Liu et al. reported the latter to be associated with larger DCIS size, high cytonuclear grade and increased ki67 proliferation index [21]. In our referral opinion experience, we have seen examples of missed foci of microinvasion in surgical specimens followed by recurrent HER2-positive invasive carcinoma with associated nodal metastasis. This highlights the importance of a thorough histological examination of high-grade DCIS to identify both microinvasion and true invasive disease. ER, PR and HER2 immunohistochemistry, however, is not performed routinely in the UK on DCIS cases as per the NHSBSP breast reporting guidelines and therefore information on receptor status of DCIS/microinvasion within the Sloane Project was only available for a minority of cases. However, the current practice is evolving, and pathologists are increasingly reporting ER/PR/HER2 on microinvasive foci as additional useful data that could guide adjuvant therapy and provide prognostic information.

The lack of consistency in the histological definition of microinvasion, particularly in the early literature may explain, at least in part, the conflicting conclusions of various studies. Lagios et al. [22] defined microinvasion as small invasive foci measuring less than 1 mm in 1982, but the term was subsequently used inconsistently, with a broad range of definitions. In 1988, Silver and Tavassoli [23] defined a microinvasive lesion as DCIS with a single focus of invasion not exceeding 2 mm in the largest dimension, or up to three foci of invasion, none of which was more than 1 mm in the greatest dimension. The former group clearly included lesions that would currently be classified as small (pT1a) invasive carcinoma.

The current strict definition and updated and clarified histological guidelines are likely to be the reasons for the reduction in the incidence of microinvasion noted over the past decade in the Breast Screening Programme Sloane data. The diagnostic criteria for the current Sloane cohort (2003–2012) included an invasive size of <1 mm within non-specialised stroma. The latter criterion was removed in the UK 2016 guidelines, in line with international guidance; in view of the difficulties in discerning non-specialised stroma within high-grade DCIS where the brisk inflammatory infiltrate is likely to obscure the stromal features. This update post-dated the Sloane inclusion period and therefore should not have impacted on the diagnostic criteria of the included lesions. As for multifocality, current pathology guidelines recommend measurement of the largest focus if multiple microinvasive foci are identified microscopically, not adding them up, with a comment on the number/multiplicity of foci of microinvasion [16] The reason for variation in the reported incidence among screening units is, however, not clear and requires further evaluation. It is unlikely to be true due to demographic differences and raises concern about a lack of reproducibility in the pathological diagnosis. Due to the focal nature of the lesion, the consistency of pathologists reporting of microinvasion has not been evaluated in previous studies, or in the UK Breast External Quality Assurance Scheme. The recent transition of the scheme to digital whole slide images will enable assessment of the consistency of reporting of focal lesions such as microinvasive carcinoma and will be valuable as an educational tool.

Superimposed on the current standard definition (foci of invasion of ≤1 mm in size), de Mascarel et al. have proposed sub-classifying microinvasion in the periductal stroma into M1 (invasive individual cells) and M2 lesions (invasive small clusters). The authors reported no nodal metastasis in patients in the M1 group whereas 10.1% of patients in the M2 group had nodal metastasis [24]. They suggested that there were two biological types of microinvasion, one (M1) behaving like DCIS and another (M2) with a greater capacity for invasive behaviour. This clearly has potential clinical relevance but requires further validation. Molecular signatures that distinguish poor prognosis DCIS that is likely to progress to invasive carcinoma/metastasise are being investigated, and recent genomic data from the Sloane cohort highlighted clonal similarities between the primary DCIS and the subsequent invasive recurrences in 75% of cases [25]. Going forward, the molecular profile may provide an attractive companion to standard histopathological examination to dissect the good prognosis microinvasive lesions from those that have the potential to frankly invade/metastasise.

To summarise, this is the second largest series of microinvasion reported to date, and the largest prospective well-characterised cohort of screen-detected DCIS and microinvasion globally. The main strengths of the study include the large cohort size, prospective nature of data collection and robustness and rigorous validation of the collected multidisciplinary data, making the results valid and generalisable. Some of the limitations of this study include the lack of information on the number of microinvasive foci and whether the diagnosis of microinvasion was made on core biopsy and/or surgical excision; although we anticipate that the majority will have been diagnosed on surgical excision specimens. The Sloane audit was a prospective audit that collected a huge amount of high-quality pathology, surgical, oncology and outcome data, but central pathology review was not required. Based on these findings, however, microinvasive breast carcinoma appears to be more aggressive than pure DCIS and is associated with higher rate of distant metastases and higher breast cancer mortality. It is also of note that subsequent invasive carcinoma recurrences were mostly Grade 3 and thus of poorer prognostic histology. We believe that a sentinel node biopsy should be considered, in a multidisciplinary approach, with appropriate discussion with patients, if the diagnosis of very small foci of invasion, akin to microinvasion, is made on core biopsy. There is no evidence, as yet, to suggest whether such patients benefit from subsequent systemic therapy. Several factors such as the number of microinvasive foci, the molecular profile specially HER2 status and possibly the genomic signature may need to be collectively considered by the multidisciplinary team and patients to tailor subsequent therapy.

## Supplementary information

aj-checklist

## Data Availability

Data are held by NHS England and NHS Improvement (formerly Public Health England). Access to the Sloane Project data from external parties is governed by consultation with the Sloane Project Steering Group and application to the breast screening research advisory committee (RAC) and NHS England and NHS Improvement. Data will subsequently only be released by NHS England and NHS Improvement to researchers under the approval and in an anonymised or depersonalised format, with a data sharing contract in place.
